# Nonlinear association between P-wave axis and lung function in adult population with COPD: evidence from a cross-sectional study

**DOI:** 10.3389/fmed.2026.1860150

**Published:** 2026-07-22

**Authors:** Na Zhang, Jijing Wang, Guihua Zhao, Ruike Yang, Liguo Mao, Jinyi Xu

**Affiliations:** 1 Department of Cardiopulmonary Function, Henan Provincial People’s Hospital of Zhengzhou University, Zhengzhou, Henan, China; 2 Functional Examination Department, Henan Provincial People’s Hospital, Northeast Henan Hospital, Puyang, China

**Keywords:** chronic obstructive pulmonary disease, cross-sectional study, electrocardiogram, lung function, P-wave axis

## Abstract

**Objective:**

This study aims to explore the correlation between the electrical axis of the P wave and lung function indicators in patients with chronic obstructive pulmonary disease (COPD) and the prognostic value of the P wave electrical axis in COPD.

**Methods:**

A retrospective analysis was performed on data from 407 participants at Henan Provincial People’s Hospital, Northeast Henan Hospital. Participants were categorized into control group (*n* = 206) and COPD group (*n* = 201). The COPD group was further divided into five subgroups (mild, moderate, moderate-severe, severe, and very severe) according to the percentage of predicted FEV1 (FEV1% pred). The study employed multiple linear and logistic regression analysis, restricted cubic splines (RCS) analysis, and subgroup analyses to examine the correlation between the P wave electrical axis and ventilation indicators.

**Results:**

COPD patients were older, predominantly male, and had higher smoking prevalence than controls (all *P* < 0.001). The analysis showed higher heart rate (81.3 ± 16.7 vs. 73.0 ± 12.3 bpm, *P* < 0.001) and P-wave axis (64.2 ± 21.9° vs. 41.5 ± 18.4°, *P* < 0.001) in COPD patients. Univariate linear regression demonstrated significant negative correlations between P-wave axis and pulmonary function indicators: FEV1 (β = −9.24, *P* < 0.001) and FEF75% (β = −14.72, *P* < 0.001). Logistic regression analysis showed that the P-wave axis was independently and positively associated with the risk of COPD (OR = 1.05, *P* < 0.001). The RCS analysis further suggests a non-linear pattern “J-shaped”, between P-wave axis and COPD risk, and beyond a specific threshold, at 61.9°, risk increased more sharply above this threshold. Notably, the steep upward trend of risk for P-wave axis > 75° was limited by sparse sample distribution and only for exploratory reference. Additionally, subgroup analysis indicates a stronger association in males than females (OR = 1.06, *P* < 0.05). Subgroup results should be interpreted cautiously due to reduced statistical power within small stratified populations.

**Conclusion:**

The P-wave axis is closely associated with pulmonary function and COPD risk, exhibiting a nonlinear “J-shaped” relationship with 61.9° (95%CI: 61.5°, 62.4°) inflection point.

## Introduction

Chronic obstructive pulmonary disease (COPD) is a prevalent respiratory condition characterized by persistent respiratory symptoms and airflow limitation, contributing to significant morbidity and mortality globally (1). Recent epidemiological studies indicate that the prevalence of COPD among individuals aged over 40 in China has reached 13.7%, underscoring its substantial public health impact. The pathophysiological manifestations of COPD extend beyond the pulmonary system, as systemic inflammation, hypoxemia, and oxidative stress often result in multi-organ involvement, with cardiovascular complications being particularly notable (2, 3). As such, early screening and quantitative assessment of electrocardiogram-detected cardiovascular changes associated with COPD are critical to the timely identification of COPD (4). This study seeks to develop a straightforward and quantifiable electrophysiological assessment tool for clinical application, enabling clinicians to preliminarily assess the severity of airflow limitation through routine electrocardiograms, particularly when pulmonary function tests are not feasible. This methodology aims to promote the early detection and management of COPD and its associated cardiovascular complications.

## Materials and methods

### Study design and population

A retrospective analysis was conducted on 2,804 clinical data collected from patients with COPD who attended the Functional Examination Department at Henan Provincial People’s Hospital Northeast Henan Hospital between September 2024 and September 2025. The control group comprised individuals with normal pulmonary function during the same time frame. Inclusion criteria for the COPD group were based on the “Guidelines for the Diagnosis and Treatment of Chronic Obstructive Pulmonary Disease (2021 revised edition)” by the Chinese Society of Respiratory Diseases, specifically defined by a post-bronchodilator FEV1/FVC less than 70%, with the exclusion of other diseases presenting similar clinical symptoms. The normal control group: Patients with all indicators of pulmonary function tests within the normal predicted values, and cardiac color Doppler ultrasound excluded organic cardiovascular lesions including left/right heart failure, pulmonary hypertension, pulmonary vascular structural abnormalities, valvular heart disease, cardiomyopathy and atrial/ventricular enlargement; subjects with any above cardiac lesions were excluded even if their lung function was within normal range (5).

Exclusion criteria encompassed acute myocardial infarction, unstable angina, or severe heart failure (NYHA functional class III–IV) within the preceding 3 months, as well as severe arrhythmias, such as atrial fibrillation, frequent premature atrial contractions, second-degree or higher atrioventricular block, and instances where the P wave could not be clearly identified. Severe uncontrolled hypertension (systolic ≥ 200 mmHg or diastolic ≥ 100 mmHg), significant thoracic or spinal deformities, severe thyroid disorders, acute stroke, active pulmonary tuberculosis, lung cancer, recent major thoracoabdominal surgeries, and recent use of heart rate or electrophysiological-affecting drugs (e.g., beta-blockers, digoxin). Among all enrolled patients, 206 were diagnosed with COPD, and 201 had normal pulmonary ventilatory function.

### Pulmonary function testing

All participants underwent pulmonary function testing using the German Jaeger Master Screen spirometer. The testing was strictly conducted in accordance with the quality control requirements of the ERS/ATS Pulmonary Function Guidelines and the Chinese Society of Respiratory Diseases Pulmonary Function Guidelines (6).

Pulmonary Function Indicator Standards: Ventilatory function indicators were assessed using the 2012 Global Lung Function Initiative (GLI) Southeast Asian reference equations for normal pulmonary function values. Other indicators used the European Chest Society (ECCS93) recommended reference values. Prior to the test, participants were instructed to rest for 15–20 min. Their height, weight, and age were recorded.

Procedure: Participants wore a nose clip and used a mouthpiece to breathe, starting with quiet breathing, then inhaling deeply to full lung capacity, and exhaling forcefully to residual volume. Quality Control: Each participant completed at least three tests meeting ATS/ERS standards, with the best result used for analysis. Measured indicators included FVC, FEV1, FEV1/FVC, FEV3, FEV3/FVC, MMEF, FEF50%, FEF75%, and percentage of predicted values. Interpretation: Two certified junior physicians independently interpreted the results, with a final review by a senior physician qualified as a pulmonary function training supervisor.

### Electrocardiogram (ECG) examination

On the same day as the pulmonary function test, participants underwent a standard 12-lead electrocardiogram in the supine position. A fully automatic 12-lead ECG machine (Nihon Kohden, Japan) was used. Parameter Settings: Paper speed: 25 mm/s, Gain: 10 mm/mV

Measured Parameters: P-wave axis (P-axis),Heart rate (HR), P-wave duration, R_*V5*_ and S_*V1*_ amplitudes. Interpretation: The ECGs were independently interpreted by a junior physician and subsequently reviewed by a senior physician (7). The inter-rater reliability ICC of P-wave axis measurement was 0.927 (95%CI: 0.901–0.948), and ICC of FEV1%pred interpretation was 0.941 (95%CI: 0.918–0.959), indicating excellent consistency between interpreters.

### Statistical analysis

Measurement data with a normal distribution were expressed as mean ± standard deviation, while non-normally distributed data were presented as median (interquartile range). Comparisons of measurement data between two groups were performed using the independent samples *t*-test or nonparametric tests, while comparisons among three or more groups were conducted using analysis of variance (ANOVA). Categorical data were expressed as frequencies and percentages, and differences between groups were analyzed using the Chi-square test. Pearson correlation analysis was used to explore the correlation between the P-wave axis and various pulmonary function parameters. A multivariable linear regression model was established to evaluate the effects of ventilatory parameters on the electrical axis. Multivariate logistic regression was used to analyze the risk of COPD. A restricted cubic spline (RCS) model to develop smooth curves for examining potential nonlinear dose-response associations between variable P-wave axis and outcome COPD risk. P-wave axis was treated as a continuous variable, incorporating four knots. Nonlinearity was assessed using a likelihood ratio test, which compared a model containing only a linear term to a model that included both linear and cubic spline terms. Missing data handling: We first evaluated missing data patterns via visual missing matrix and Little’s Missing Completely at Random (MCAR) test. All variables in this study had missing proportion < 1.2%, and Little’s test *P* = 0.621, confirming data missing completely at random. Cases with any missing key indicators (pulmonary function indices, ECG P-wave axis, covariates including age, gender, smoking, BMI) were directly excluded by listwise deletion before regression, spline and subgroup analyses; no multiple imputation was applied due to extremely low missing rate and MCAR pattern. Verification of statistical model assumptions: Before statistical modeling, all model assumptions were strictly verified. Independent samples *t*-test & one-way ANOVA: Shapiro–Wilk normality test for continuous variables; Levene’s test for homogeneity of variance. Non-normal distributed variables used nonparametric Kruskal–Wallis test for intergroup comparison. Multivariate linear regression: Residual normality Q–Q plot, homoscedasticity residual scatter plot, variance inflation factor (VIF < 5) to exclude multicollinearity. Binary logistic regression: Linear relationship between continuous covariates and logit outcome via Box-Tidwell test; absence of influential outliers via Cook’s distance; Hosmer–Lemeshow goodness-of-fit test. Restricted cubic spline: Likelihood ratio test to compare linear vs. spline model for nonlinearity; visual inspection of spline smooth curve to avoid overfitting. All assumption test results were pre-specified in the analytic workflow before formal data output. Model fitting evaluation indicators: Model fitting metrics were calculated for all regression models: Linear regression: Adjusted *R*^2^, residual standard error; Logistic regression: Akaike Information Criterion (AIC), Bayesian Information Criterion (BIC), Hosmer–Lemeshow goodness-of-fit *χ*^2^ statistic; RCS spline model: Likelihood ratio test for nonlinearity, AIC for optimal knot selection (4 knots pre-specified). Stratified analysis by gender was pre-specified *a priori* in the original research protocol before data extraction, based on two established clinical evidences: (1) gender differences in COPD susceptibility and tobacco toxicity response reported in previous population cohorts; (2) anatomical thoracic and cardiac positional differences between male and female that may modify P-wave axis deviation degree. Gender interaction test was performed to quantify effect modification, while age, BMI, smoking subgroups were exploratory *post hoc* stratified analyses only for supplementary reference. Clarification for cross-sectional logistic regression. This study is a cross-sectional retrospective case-control study; binary logistic regression was applied to estimate cross-sectional odds ratio (OR) reflecting the concurrent association between continuous P-wave axis and prevalent COPD diagnosis, rather than predicting incident COPD requiring longitudinal follow-up data. OR in this cross-sectional setting represents the odds of having COPD per 1° increment of P-wave axis among contemporaneous participants, which is a standard epidemiological analytical method for cross-sectional prevalence data. All analyses in this study were conducted using R software (version 3.3.2^1^, The R Foundation) and Free Statistics software (version 2.3, Free Statistics software). A two-sided *P*-value of less than 0.05 was considered statistically significant.

## Result

### Baseline characteristics of study participants

A total of 407 participants were included, comprising 206 healthy controls and 201 patients with COPD (Table 1). There were significant differences between the two groups in age, gender, and smoking history. The control group ranged from 18.0 to 81.0 years (mean 52.3 ± 16.0), with 45.6% male (94/206) and 28.6% (59/206) with a history of smoking. The COPD group ranged from 22.0 to 87.0 years (mean 65.3 ± 13.8), with 70.1% male (141/201) and 53.7% (108/201) with a history of smoking. Pulmonary function indices were significantly lower in the COPD group (all *P* < 0.001). Regarding ECG findings, the control group had a heart rate of 73.0 ± 12.3 bpm and a P-wave axis of 41.5 ± 18.4°. The COPD group showed a higher heart rate (81.3 ± 16.7 bpm) and an increased P-wave axis (64.2 ± 21.9°) compared with controls (both *P* < 0.001).

**TABLE 1:** Baseline characteristics of the participants (*n* = 407).

Variables	Total (*n* = 407)	Control (*n* = 206)	COPD (*n* = 201)	*P-*value
Gender, *n* (%)				<0.001
Male	235 (57.7)	94 (45.6)	141 (70.1)	
Female	172 (42.3)	112 (54.4)	60 (29.9)	
Age (years)	58.7 ± 16.3	52.3 ± 16.0	65.3 ± 13.8	<0.001
BMI (kg/m^2^)	25.4 ± 4.8	25.8 ± 4.1	25.1 ± 5.4	0.146
Smoking status, *n* (%)				<0.001
No	240 (59.0)	147 (71.4)	93 (46.3)	
Yes	167 (41.0)	59 (28.6)	108 (53.7)	
FVC (L)	3.0 ± 1.0	3.4 ± 1.0	2.6 ± 1.0	<0.001
FVC pred (%)	95.8 ± 22.9	107.8 ± 14.5	83.5 ± 23.4	<0.001
FEV1 (L)	2.2 ± 1.0	2.8 ± 0.8	1.5 ± 0.8	<0.001
FEV1pred (%)	85.8 ± 30.8	107.9 ± 14.3	63.0 ± 26.3	<0.001
FEV3 (L)	2.7 ± 1.1	3.3 ± 0.9	2.1 ± 0.9	<0.001
FEV3/FVC (%)	90.7 ± 11.6	97.7 ± 2.4	83.4 ± 12.7	<0.001
FEV1/FVC (%)	70.7 ± 17.3	82.7 ± 5.1	58.3 ± 16.6	<0.001
FEV1/FVCpred (%)	91.2 ± 21.0	105.5 ± 6.2	76.6 ± 20.7	<0.001
FEF75 (L)	0.7 (0.3, 1.2)	1.2 (0.9, 1.5)	0.3 (0.2, 0.6)	<0.001
FEF75pred (%)	60.5 (33.5, 82.3)	80.3 (66.8, 97.2)	32.8 (19.7, 49.3)	<0.001
FEF50 (L)	2.4 (0.9, 3.5)	3.4 (2.7, 4.2)	0.8 (0.4, 1.7)	<0.001
FEF50pred (%)	61.6 ± 37.5	90.0 ± 21.2	32.5 ± 26.6	<0.001
MMEF (L/s)	1.9 (0.7, 2.8)	2.8 (2.1, 3.4)	0.7 (0.4, 1.4)	<0.001
MMEFpred (%)	61.2 ± 34.4	88.3 ± 19.6	33.4 ± 21.8	<0.001
HR (bpm)	77.1 ± 15.2	73.0 ± 12.3	81.3 ± 16.7	<0.001
P-wave duration (ms)	100.6 ± 12.1	99.7 ± 10.2	101.3 ± 11.9	0.762
P-wave axis (°)	52.6 ± 23.1	41.5 ± 18.4	64.2 ± 21.9	<0.001
RV5 (mV)	1.5 ± 0.6	1.5 ± 0.6	1.7 ± 0.3	0.164
SV1 (mV)	0.8 ± 0.5	0.9 ± 0.4	0.8 ± 0.5	0.494

Continuous data: mean ± SD (normal) / median (IQR) (skewed); categorical data: *n* (%). Inter-rater reliability ICC: P-wave axis measurement ICC = 0.927 (95%CI 0.901–0.948); FEV1%pred interpretation ICC = 0.941 (95%CI 0.918–0.959), indicating excellent observer agreement. Mean ± SD for normally distributed continuous variables, Median (IQR) for non-normally distributed continuous variables, (%) for categorical variables. *FVC* forceful lung volume, *FEV1* forceful expiratory volume in 1 s, *FEV1pred* predicted forced expiratory volume in 1 s (%), *FEV3* forced expiratory volume in 3 s, *FEV3/FVC* ratio of forced expiratory volume in 3 s to forced vital capacity, *FEF75* forced expiratory flow at 75% of FVC, *FEF50* forced expiratory flow at 50% of FVC, *MMEF* maximal mid-expiratory flow, FEF 50% forceful expiratory flow in 50% of FVC, *HR* heart rate, *RV5* V5 lead R-wave amplitude, *SV1*,V1 lead S-wave amplitude.

### Comparison of baseline data and clinical characteristics in COPD subgroups

**FIGURE 1:**
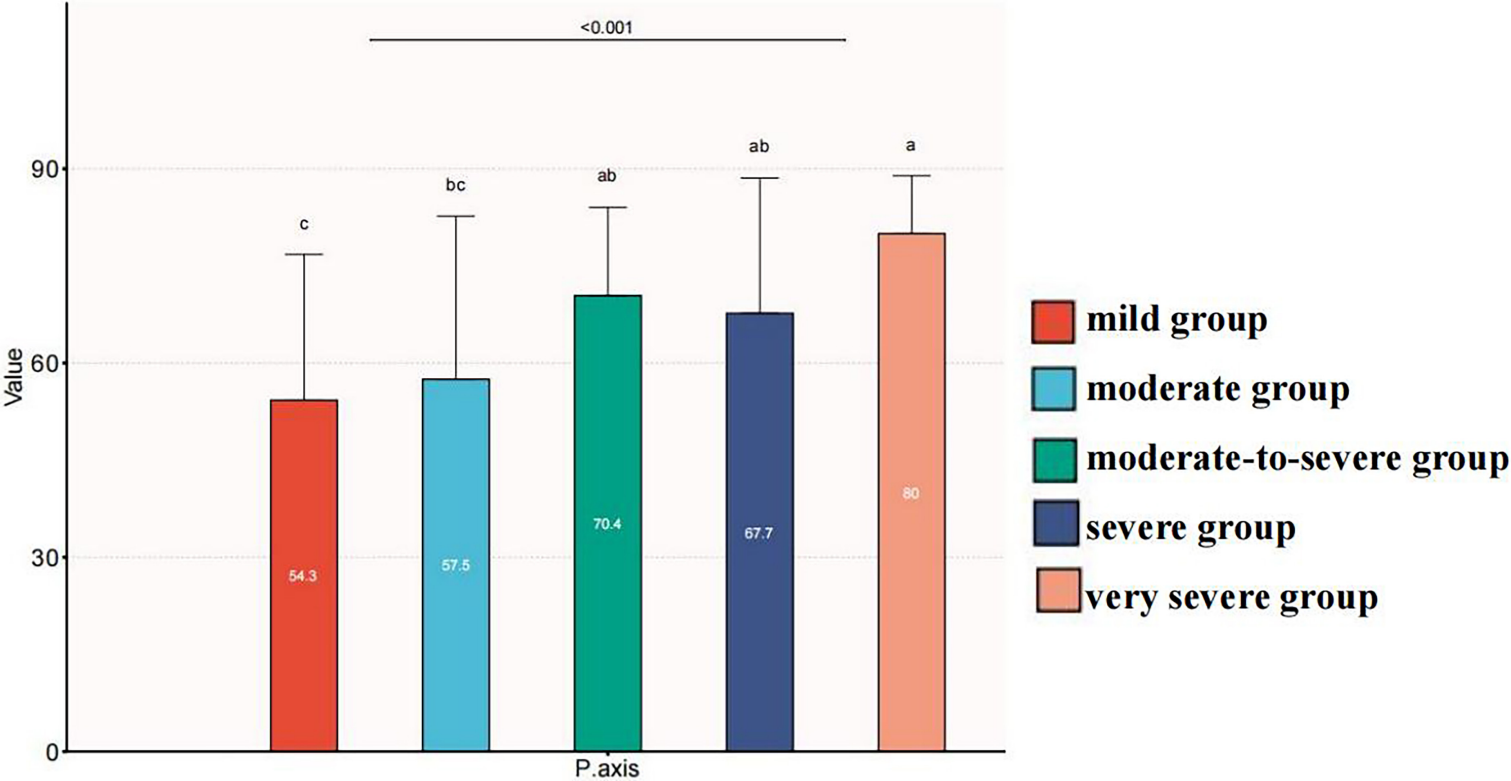
Comparison of P-wave axis across five COPD severity subgroups classified by FEV1% predicted. One-way ANOVA was applied for crude intergroup comparison, followed by pairwise *post hoc* test; superscript letters (a/b/c/ab/bc) denote significant between-group differences (overall *P* < 0.001). Note: ANOVA only presents unadjusted crude group differences; multivariate regression in subsequent analysis further adjusted age, gender, BMI and smoking to quantify independent correlation.

Chronic obstructive pulmonary disease patients were divided into five subgroups based on the percentage of predicted FEV1 (%pred): Mild: FEV1% pred ≥ 70%,Moderate: 60%–69%,Moderate to Severe: 50%–59%,Severe: 35%–49%,Very Severe: < 35%. Baseline data and clinical characteristics of the five COPD subgroups are shown in Table 2. There were significant differences in P-wave axis, age, and heart rate across the groups (Figure 1), while no significant differences were observed in sex, BMI, smoking history, P-wave duration, RV5 amplitude, or SV1 amplitude. One-way ANOVA was used for crude intergroup descriptive comparison to visualize overall between-group disparity of P-wave axis across COPD severity grades; subsequent regression models adjusted confounders to quantify independent correlation, forming a stepwise analytical system without logical conflict.

**TABLE 2:** Comparison of baseline characteristics between groups of COPD participants.

Variables	Total (*n* = 201)	Mild group (*n* = 75)	Moderate group (*n* = 28)	Moderate-to-severe group (*n* = 24)	Severe group (*n* = 32)	Very severe group (*n* = 42)	*P*
Gender, *n* (%)							0.105
Male	141 (70.1)	47 (62.7)	17 (60.7)	19 (79.2)	23 (71.9)	35 (83.3)	
Female	60 (29.9)	28 (37.3)	11 (39.3)	5 (20.8)	9 (28.1)	7 (16.7)	
Age (years)	65.3 ± 13.8	59.9 ± 16.6	66.2 ± 14.1	70.4 ± 9.7	68.6 ± 9.3	68.9 ± 9.3	<0.001
BMI (kg/m^2^)	25.1 ± 5.4	26.2 ± 4.6	25.7 ± 8.6	24.8 ± 4.3	25.3 ± 5.0	22.5 ± 4.1	0.008
Smoking status, *n* (%)							0.503
No	93 (46.3)	39 (52)	14 (50)	8 (33.3)	15 (46.9)	17 (40.5)	
Yes	108 (53.7)	36 (48)	14 (50)	16 (66.7)	17 (53.1)	25 (59.5)	
FVC (L)	2.6 ± 1.0	3.2 ± 1.0	2.5 ± 0.7	2.5 ± 0.7	2.1 ± 0.7	1.9 ± 0.6	<0.001
FVC pred (%)	83.5 ± 23.4	101.6 ± 20.0	87.1 ± 18.0	81.3 ± 12.2	71.2 ± 14.6	59.4 ± 13.5	<0.001
FEV1 (L)	1.5 ± 0.8	2.2 ± 0.7	1.5 ± 0.5	1.3 ± 0.3	1.0 ± 0.3	0.7 ± 0.2	<0.001
FEV1pred (%)	63.0 ± 26.3	89.5 ± 15.6	68.8 ± 11.6	57.5 ± 5.4	44.7 ± 6.1	29.1 ± 6.7	<0.001
FEV3 (L)	2.1 ± 0.9	2.9 ± 0.9	2.2 ± 0.6	2.0 ± 0.5	1.6 ± 0.5	1.2 ± 0.3	<0.001
FEV3/FVC (%)	83.4 ± 12.7	92.3 ± 5.7	88.5 ± 7.2	83.2 ± 9.4	79.4 ± 11.2	67.4 ± 10.8	<0.001
FEV1/FVC (%)	58.3 ± 16.6	71.4 ± 9.4	63.5 ± 10.9	56.0 ± 10.2	51.2 ± 13.4	38.3 ± 11.7	<0.001
FEV1/FVCpred (%)	76.6 ± 20.7	92.5 ± 11.8	83.2 ± 13.2	74.7 ± 13.4	67.9 ± 17.4	51.6 ± 14.1	<0.001
FEF75 (L)	0.3 (0.2, 0.6)	0.6 (0.5, 0.8)	0.3 (0.3, 0.5)	0.3 (0.2, 0.4)	0.2 (0.2, 0.3)	0.1 (0.1, 0.2)	<0.001
FEF75pred (%)	32.8 (19.7, 49.3)	49.1 (39.3, 61.0)	37.9 (28.3, 47.8)	26.5 (23.2, 37.8)	20.9 (16.5, 25.3)	14.2 (11.8, 18.8)	<0.001
FEF50 (L)	0.8 (0.4, 1.7)	2.0 (1.3, 2.5)	1.0 (0.8, 1.4)	0.8 (0.6, 0.9)	0.5 (0.4, 0.6)	0.3 (0.2, 0.4)	<0.001
FEF50pred (%)	32.5 ± 26.6	55.4 ± 26.6	34.2 ± 21.6	22.2 ± 7.1	16.3 ± 6.6	8.8 ± 2.9	<0.001
MMEF (L/s)	0.7 (0.4, 1.4)	1.6 (1.1, 2.0)	0.8 (0.6, 1.1)	0.6 (0.6, 0.8)	0.5 (0.4, 0.6)	0.3 (0.2, 0.3)	<0.001
MMEFpred (%)	33.4 ± 21.8	54.1 ± 16.1	36.3 ± 18.8	25.4 ± 8.1	18.7 ± 7.4	10.6 ± 3.3	<0.001
HR (bpm)	81.3 ± 16.7	74.6 ± 12.7	81.0 ± 16.0	79.2 ± 13.7	85.2 ± 18.7	91.8 ± 17.8	<0.001
P-wave duration (ms)	101.3 ± 13.9	99.7 ± 8.1	100.2 ± 11.0	102.8 ± 7.9	99.8 ± 7.9	96.2 ± 10.3	0.57
P-wave axis (°)	64.2 ± 21.9	54.3 ± 22.5	57.5 ± 25.2	70.4 ± 13.6	67.7 ± 20.9	80.0 ± 9.0	<0.001
RV5 (mV)	1.5 ± 0.6	1.6 ± 0.6	1.4 ± 0.6	1.6 ± 0.7	1.4 ± 0.5	1.3 ± 0.6	0.078
SV1 (mV)	0.8 ± 0.5	0.9 ± 0.4	0.9 ± 0.5	0.6 ± 0.4	0.9 ± 0.6	0.7 ± 0.6	0.05

Baseline indicators stratified by COPD severity subgroups. One-way ANOVA/Kruskal–Wallis test for intergroup crude comparison, unadjusted for confounders. Mean ± SD for Normally distributed continuous variables, Median (IQR) for non-normally distributed continuous variables, (%) for categorical variables. FVC forceful lung volume, FEV1 forceful expiratory volume in 1 s, FEV1pred predicted forced expiratory volume in 1 s (%), FEV3 forced expiratory volume in 3 s, *FEV3/FVC* ratio of forced expiratory volume in 3 s to Forced Vital Capacity, *FEF75* Forced Expiratory Flow at 75% of FVC, *FEF50* forced expiratory flow at 50% of FVC, *MMEF* maximal mid-expiratory flow, FEF 50% forceful expiratory flow in 50% of FVC, *HR* heart rate, *RV5* V5 lead R-wave amplitude, *SV1*,V1 lead S-wave amplitude.

### Linear regression analysis of P-wave axis and pulmonary parameters

The univariate linear regression analysis of the P-wave axis and pulmonary function parameters is shown in Table 3. Results indicated a significant negative correlation between the P-wave axis and FVC, FEV1, FEV1/FVC, FEV3, FEV3/FVC, MMEF, FEF50, FEF75, and their corresponding predicted percentages (all *P* < 0.001). Notably, the P-wave axis was significantly negatively correlated with FEV1 (β = −9.24, *P* < 0.001) and FEF75 (β = −14.72, *P* < 0.001).

**TABLE 3:** Univariate and multivariable linear regression analysis of P-wave axis and pulmonary function parameters.

Variable	Crude.β (95%CI)	Crude.*P* value	Adj.β (95%CI)	Adj.*P* value
FVC	−4.77 (−6.88∼−2.67)	<0.001	−4.8 (−7.34∼−2.27)	<0.001
FVC pred	−0.34 (−0.44∼−0.25)	<0.001	−0.23 (−0.32∼−0.13)	<0.001
FEV1	−9.24 (−11.26∼−7.21)	<0.001	−8.69 (−11.15∼−6.22)	<0.001
FEV1pred	−0.37 (−0.44∼−0.31)	<0.001	−0.29 (−0.35∼−0.22)	<0.001
FEV3	−7.11 (−9.05∼−5.18)	<0.001	−6.91 (−9.23∼−4.58)	<0.001
FEV3/FVC	−1.11 (−1.27∼−0.94)	<0.001	−0.89 (−1.08∼−0.7)	<0.001
FEV1/FVC	−0.77 (−0.88∼−0.66)	<0.001	−0.65 (−0.78∼−0.52)	<0.001
FEV1/FVC pred	−0.61 (−0.7∼−0.53)	<0.001	−0.5 (−0.6∼−0.4)	<0.001
FEF75	−14.72 (−17.91∼−11.52)	<0.001	−12.01 (−16.05∼−7.97)	<0.001
FEF75 pred	−0.09 (−0.12∼−0.05)	<0.001	−0.06 (−0.09∼−0.03)	<0.001
FEF50	−7 (−8.24∼−5.77)	<0.001	−5.9 (−7.34∼−4.46)	<0.001
FEF50 pred	−0.32 (−0.37∼−0.27)	<0.001	−0.25 (−0.31∼−0.19)	<0.001
MMEF	−8.25 (−9.76∼−6.75)	<0.001	−7.12 (−8.96∼−5.29)	<0.001
MMEFpred	−0.36 (−0.41∼−0.3)	<0.001	−0.28 (−0.35∼−0.22)	<0.001

Adjusted *R*^2^ of full multivariate model = 0.312, no multicollinearity (all VIF < 5). Adj: adjusted age, gender, BMI, smoking status. FVC forceful lung volume, FEV1 forceful expiratory volume in 1 s, FEV1pred predicted forced expiratory volume in 1 s (%), FEV3 Forced Expiratory Volume in 3 s, FEV3/FVC ratio of forced expiratory volume in 3 s to forced vital capacity, FEF75 forced expiratory flow at 75% of FVC, FEF50 forced expiratory flow at 50% of FVC, MMEF maximal mid-expiratory flow, FEF 50% forceful expiratory flow in 50% of FVC, HR heart rate, RV5 V5 lead R-wave amplitude, SV1,V1 lead S-wave amplitude.

### Logistic regression analysis of ECG parameters and COPD

The univariate logistic regression analysis is shown in Table 4. The results indicated that heart rate (OR = 1.04, *P* < 0.001) and P-wave axis (OR = 1.06, *P* < 0.001) were positively correlated with COPD. After adjusting for traditional risk factors such as gender, age, BMI, and smoking history, the P-wave axis was independently and positively associated with the risk of COPD (OR = 1.05, *P* < 0.001).(Table 4). Each 1-degree increase in P-wave axis was independently associated with higher odds of COPD (adjusted OR = 1.05, 95%CI [1.02–1.08], *P* < 0.001).

**TABLE 4:** Logistic regression analysis of electrocardiographic parameters and COPD.

Variable	Model 1		Model 2	
	OR (95%CI)	*P*-value	OR (95%CI)	*P*-value
HR	1.04 (1.03∼1.06)	**<0.001**		
P-wave duration	1 (1∼1)	0.841		
P-wave axis	**1.06 (1.04∼1.07)**	**<0.001**	**1.05 (1.03∼1.06)**	**<0.001**
RV5	0.79 (0.56∼1.1)	0.165		
SV1	0.86 (0.57∼1.31)	0.494		

Binary logistic regression for cross-sectional prevalent COPD (not incident follow-up outcome). Model 1 = unadjusted crude OR; Model 2 = fully adjusted OR (age, gender, BMI, smoking). Hosmer–Lemeshow goodness-of-fit χ^2^ = 7.12, *P* = 0.416 (good model fit). AIC = 216.3, BIC = 247.9. Bold values indicate statistically significant differences (*p* < 0.05).

### Nonlinear association analysis of ECG parameters and COPD

**FIGURE 2:**
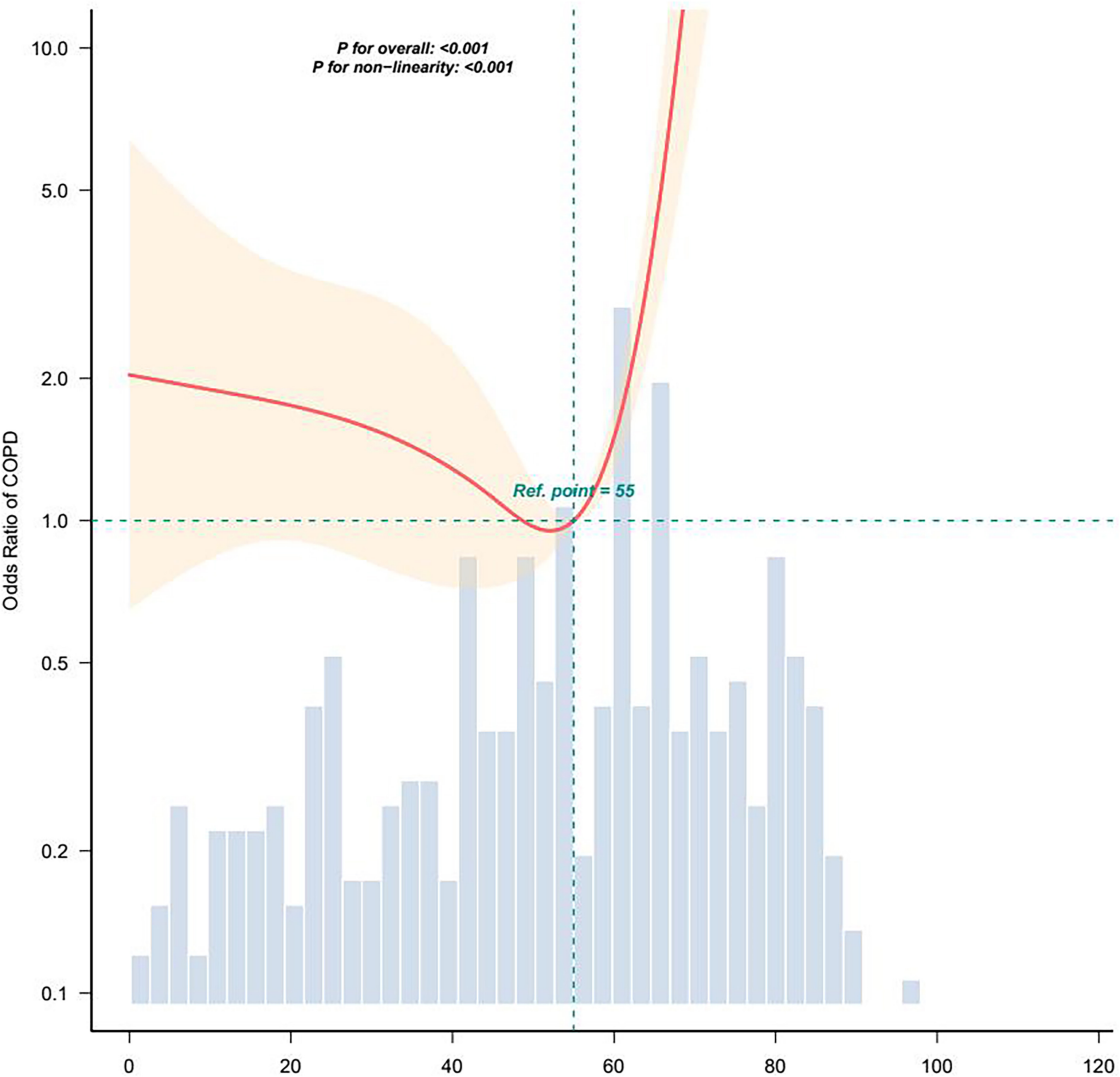
Restricted cubic spline (RCS) analysis for nonlinear association between P-wave axis and cross-sectional COPD odds ratio (OR). Red smooth curve = adjusted OR curve; light beige shadow = 95% confidence interval; grey histogram = sample distribution density across P-wave values; horizontal dashed line = reference OR = 1 at P-axis = 55; vertical dashed line = inflection point at 61.9°. Overall association *P* < 0.001, nonlinearity likelihood ratio test *P* < 0.001. Critical caution: The steep upward trend of OR for P-axis>75ed smooth curve = adjusted OR curve; light beige shadow = 95% confidence interval; grey histogram = ratory reference only and cannot support definitive clinical inference; reliable risk elevation trend is only valid within 61.9°–75°.

Restricted cubic splines analysis was used to assess the relationship between P-wave axis and COPD risk, including inflection point analysis. The results revealed a significant nonlinear association (*P* < 0.001) (Figure 2). The histogram under the spline curve reflects sample distribution density across P-wave axis values. Samples with P-wave axis > 75° account for only 8.1% of total participants (*n* = 33), corresponding to sparse right tail data. When the P-wave axis was below 61.9° (95%CI: 61.5°, 62.4°), the increase in disease risk was relatively gradual. However, when the P-wave axis exceeded 61.9°, the risk increased more sharply, and the slope of the curve became steeper (Table 5). Figure 2 legend: Restricted cubic spline curve for P-wave axis and COPD odds ratio; Note: The steep odds ratio rise for P-wave axis > 75° is exploratory due to sparse sample distribution in this range.

**TABLE 5:** Threshold effect analysis of P-wave axis and COPD.

P-wave axis	*n*	Breakpoint/OR (95%CI)	*P*-value
61.905	402	61.905 (61.456, 62.354)	0.4777
<61.9	238	0.993 (0.975∼1.012)	
≥61.9	164	1.524 (1.265∼1.834)	**<0.001**
Likelihood ratio test			<0.001

Threshold effect analysis of P-wave axis and COPD risk adjusted for covariates. Inflection point = 61.9°; *n* = 238 below cutoff, *n* = 164 above cutoff. Subjects with P-axis > 75° (*n* = 33) were excluded from threshold quantitative estimation due to insufficient sample density. Bold values indicate statistically significant differences (*p* < 0.05).

### Subgroup analysis: effect of different gender on the association

**FIGURE 3:**
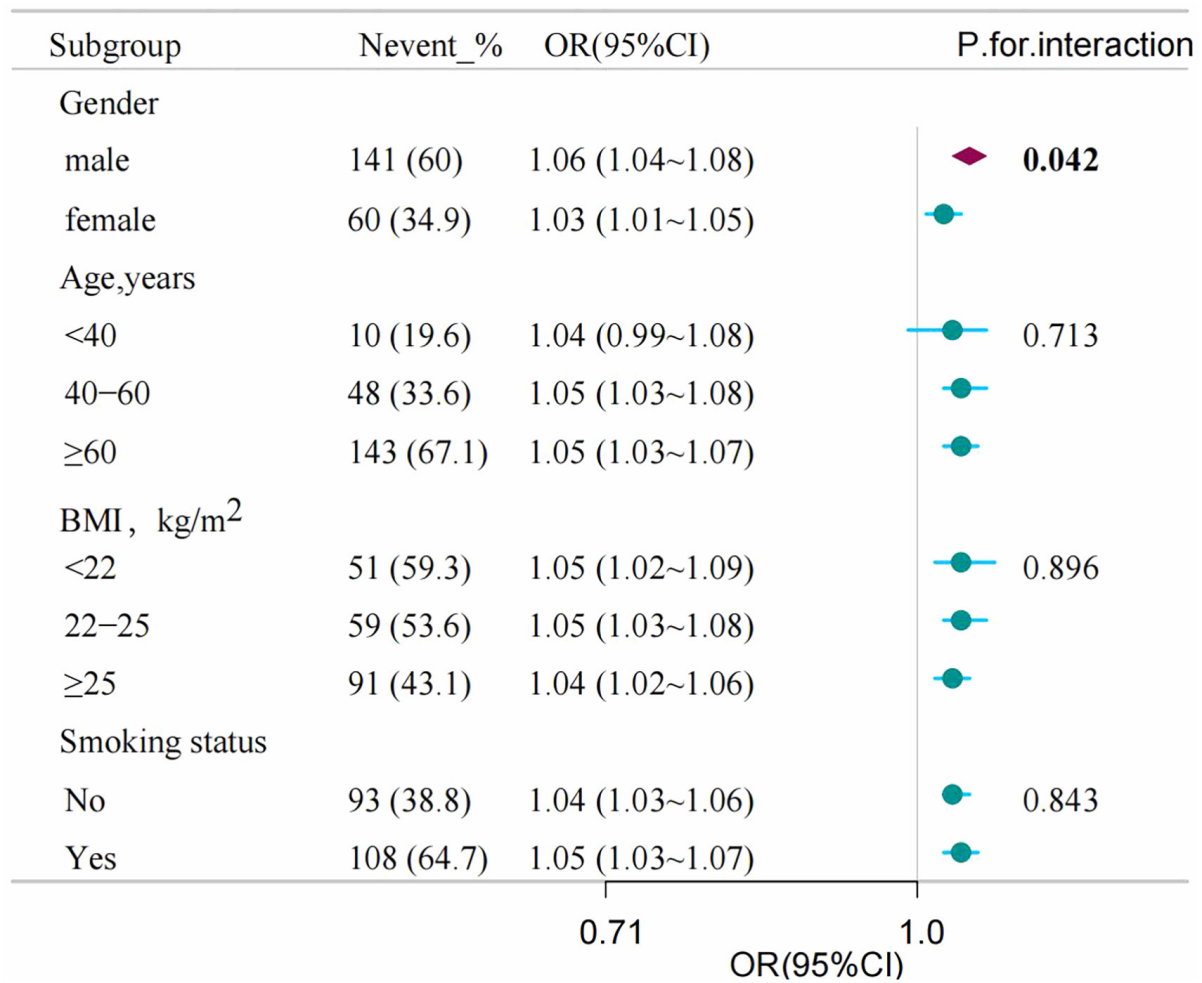
Stratified subgroup analysis of adjusted OR for COPD risk per 1° increment of P-wave axis. Gender stratification was defined *a priori* in study protocol; age, BMI and smoking subgroups were exploratory *post hoc* analyses. P for interaction (gender) = 0.042. Multiple stratifications reduced sample size and statistical power within subgroups; effect sizes from small subgroups (female COPD *n* = 60) should be interpreted cautiously and not used as diagnostic cutoffs.

Gender-stratified analysis (Figure 3) showed that the association between P-wave axis and COPD was stronger in males (OR = 1.06, *P* < 0.05) compared with females. Multiple subgroup stratifications reduced sample size within each stratum and lowered statistical power; effect sizes of small subgroups (especially female COPD patients *n* = 60) should not be generalized as definitive diagnostic thresholds.

## Discussion

The prevalence of cardiovascular comorbidities, including ischemic heart disease, arrhythmia, and heart failure, in patients with COPD can reach up to 50%, with such comorbidities representing independent risk factors for acute exacerbations and mortality (8, 9). This study focuses on the relationship between P-wave axis verticalization on electrocardiography (ECG) and COPD, as well as pulmonary ventilation indices. In the pathophysiological progression of COPD, lung hyperinflation and emphysema are central processes. Small airway remodeling and decreased alveolar elastic recoil lead to significant increases in total lung capacity and residual volume. These anatomical changes not only compress adjacent vasculature but also alter cardiac spatial orientation within the thorax. Lung expansion forces the diaphragm downward and flattens it, causing the heart to rotate clockwise, shifting from a horizontal or semi-vertical to a more vertical position. This positional alteration is reflected in surface ECG vector changes, particularly in the P-wave axis, which represents atrial depolarization (10, 11) ECG, being inexpensive, noninvasive, and widely accessible, offers irreplaceable advantages in primary care and large-scale epidemiological screening. Previous studies have reported “pulmonary P-waves” or rightward P-wave axis deviation in pulmonary heart disease, but most are limited to qualitative descriptions and focus on end-stage COPD (12). Systematic, large-sample investigations quantifying the relationship between P-wave axis deviation and pulmonary ventilation parameters remain lacking. In particular, whether subtle P-wave axis deviations in patients with mild to moderate airflow limitation can serve as an early indicator of pulmonary dysfunction remains undetermined.

Research results indicate that male gender, age, and smoking history are high-risk factors for COPD. The heart rate in the COPD group is faster than that in the normal group, and the proportion of arrhythmias, such as premature beats, tachycardia, and atrial fibrillation, is higher, with the P-wave axis value also increased. Although the prevalence of COPD has decreased in recent years in China, the crude incidence rate continues to rise, particularly among the elderly (13). Increasing evidence shows that tobacco exposure is the most important independent risk factor for COPD. Çolak et al. (14) found that even with a smoking history of fewer than 10 pack-years, 23% of smokers progressed to COPD within 5 years. Long-term follow-up also revealed that the acute exacerbation risk in this population increased by 1.83 times, and the overall mortality risk increased by 31%, with a clear dose-response relationship. Further studies indicate that gender is a key factor influencing susceptibility to tobacco toxicity. Data from Çolak et al. (15) show that women have a stronger biological susceptibility to tobacco toxicity. In heavy smokers with ≥ 50 pack-years, women had a 41.6-fold higher risk of acute exacerbation and an 11.1-fold higher risk of respiratory-related mortality compared to never-smokers, both significantly higher than men (23.7-fold and 5.66-fold, respectively). Goudis et al. (16) demonstrated that COPD is an independent risk factor for cardiovascular disease, with abnormal ECG indicators and arrhythmias such as premature beats significantly worsening the prognosis of COPD patients. In chronic obstructive pulmonary disease, rightward shift of the P-axis, typically approaching +60°, is commonly observed due to right atrial remodeling caused by secondary pulmonary hypertension. This results in a larger P-wave amplitude in lead III than in lead I, with predominantly negative P-waves. Multiple studies have confirmed that an increased P-wave axis can serve as a sensitive marker for potential COPD (17). COPD patients may exhibit specific ECG changes, with P-wave vector verticalization, prolonged QRS duration, and poor R-wave progression in the precordial leads considered the most valuable markers (18). Kulirova et al. found that patients with prognostically significant ECG abnormalities had a 2.537-fold higher risk of death within 5 years compared to those with normal ECGs (19). In the COPD population, patients with normal ECGs had a better prognosis than those with abnormal ECGs of prognostic significance, suggesting that ECG may be a valuable tool for predicting mortality risk in these patients. Changes in the ECG vector precede morphological changes in ventricular wall thickness, and the P-wave axis serves as a more sensitive early indicator of impaired pulmonary and cardiac function in COPD.

The results showed that as the degree of pulmonary ventilatory obstruction worsened, there were statistically significant differences in FVC, FEV1, FEV1/FVC, FEV3, FEV3/FVC, MMEF, FEF50, FEF75, and their corresponding predicted values, heart rate, and P-wave axis. Pairwise comparisons between the P-wave axis groups revealed statistically significant differences between the mild group and the moderate, severe, and very severe groups; the moderate group showed a significant difference from the severe group, but no significant differences were found between the moderate, severe, and very severe groups. Rusinowicz T et al. reported that Exacerbation of COPD was diagnosed in 152 patients and the prevalence of arrhythmias in this group of patients was 97%. The commonest arrhythmia was ventricular premature beats (VPB) – 88.8%, followed by supraventricular premature beats (SPB) – 56.5%. Permanent atrial fibrillation accounted for 30.3% and paroxysmal atrial fibrillation (PAF) for 12.5%. Supraventricular tachycardia (SVT) was noted in 34.2% patients and ventricular tachycardia in 25.6% (20). Alter et al. (10) assessed disease severity using the first second forced expiratory volume (FEV1) and the FEV1/FVC, confirming that the P-wave axis showed a significant increasing trend in GOLD staging. Otake S et al. (21, 22) found that COPD patients with P-wave axis > 75° had more severe airflow limitation than those with P-wave axis ≤ 75°. Patients with P-wave axis > 75° exhibited significantly higher total COPD assessment test scores, as well as higher activity and impact scores on the St. George’s Respiratory Questionnaire, compared to those with P-wave axis ≤ 75°. Additionally, in the 1- and 3-year follow-ups, patients with P-wave axis > 75° had a significantly higher incidence of acute exacerbations than those with P-wave axis ≤ 75°. The P-wave axis value is closely related to the severity of COPD.

This study found that the P-wave axis was negatively correlated with FVC, FEV1, FEV1/FVC, FEV3, FEV3/FVC, MMEF, FEF50, FEF75, and their corresponding predicted percentages, with a particularly significant correlation observed with FEF75, an indicator of small airway function. This may be because the small airways are the primary site of early COPD lesions, and increased resistance leading to elevated functional residual capacity exerts a more direct compressive effect on cardiac position than damage to large airways. As early as 1973, studies reported correlations between the P-wave axis and FEV1 percent predicted, FEV1/FVC, and residual volume fraction (RV/TLC) (23). Recent studies have demonstrated associations between the P-wave axis and peak expiratory flow (PEF) (24) as well as GOLD staging (25). Li et al. (22) followed 7,501 patients and found that cardiovascular patients with a more vertical P-wave axis had higher cardiovascular and all-cause mortality. Chhabra et al. (26) investigated both pulmonary function and CT imaging of emphysema, showing that a vertical P-wave axis was significantly positively correlated with quantitative CT emphysema and CT emphysema scores (VSE) in COPD/emphysema patients, and significantly negatively correlated with FEV1, particularly in patients with predominant lower-lobe emphysema, likely due to greater diaphragmatic descent. Chhabra L et al. reported that Prevalence of emphysema in patients with vertical P-axis was strikingly higher than in the control group: 85% vs. 4.4%. The sensitivity and specificity of vertical P-axis for diagnosing emphysema was 94.76% and 86.47%, respectively. Vertical P-axis and forced expiratory volume (FEV1) were inversely correlated (Pearson correlation coefficient = −0.683). Prevalence of severe COPD was strikingly higher in patients with P-axis > 75° as compared to the group with P-axis 60°–75°: 96.3% vs. 4.6%. Close to 80% of the emphysema patients with P-axis > 85° had very severe disease (FEV1 < 30%). P-axis verticalisation is highly effective for screening emphysema and degree of verticalisation provides a gross quantification of the disease (27). With disease progression and worsening airflow limitation, P-wave axis verticalization became more pronounced. In summary, frontal P-wave axis verticalization is not only the most characteristic and sensitive ECG manifestation in COPD/emphysema patients but also closely related to disease severity, particularly small airway involvement, highlighting its potential value for rapid COPD screening.

The P-wave axis is significantly positively correlated with the risk of incident COPD. Employing restricted cubic splines (RCS) to analyze the association between the P-wave axis and COPD risk, alongside inflection point analysis, has demonstrated a significant nonlinear relationship. Specifically, when the P-wave axis is below 61.9° (95%CI: 61.5°, 62.4°), the increase in disease risk is relatively modest; however, beyond this threshold, both the risk increment and the slope of the curve increase sharply, resulting in a “J-shaped” association. This finding provides a clear quantitative threshold for preliminary COPD screening using electrocardiography (ECG). Stratified analysis by sex showed a significant interaction effect (P for interaction = 0.042) on the association between P-wave axis and COPD risk: per 1° increase in P-wave axis, the adjusted OR was 1.06 (95% CI: 1.04–1.08) in males (*n* = 141) and 1.03 (95% CI: 1.01–1.05) in females (*n* = 60), with all models adjusted for age, smoking status, BMI, and other prespecified confounders consistent with the main analysis. The stronger association between P-wave axis and COPD risk in male participants observed in this cross-sectional dataset may be related to gender disparities in smoking exposure and thoracic anatomical characteristics reported in prior literature; mechanistic explanations cannot be confirmed without direct thoracic/cardiac imaging measurements in the current cohort, and relevant pathways require dedicated prospective research to validate.

## Conclusion

The degree of verticalization of the P-wave axis on routine ECG is closely correlated with the degree of pulmonary ventilation impairment in COPD patients, and exhibits a “J”-shaped nonlinear association with disease risk, with 61.9° as the inflection point. As a simple, cost-effective, widely accessible quantitative electrophysiological indicator, the P-wave axis can act as an auxiliary preliminary reference for airflow limitation severity evaluation and tentative COPD screening when pulmonary function testing is unavailable; subgroup stratified results should be interpreted cautiously due to reduced statistical power in small subgroups, and large prospective cohorts are required to validate its precise clinical cutoff values for diagnosis and risk stratification, with promising auxiliary clinical application value.

## Study limitations

Although this study highlights the significance of the P-wave axis in COPD assessment, several limitations exist. First, as a retrospective study, it cannot establish a definitive causal relationship; prospective cohort studies are needed to determine whether verticalization of the P-wave axis can predict future acute exacerbations of COPD. Second, echocardiographic data were not included, precluding direct comparisons between the P-wave axis and right ventricular pressure or anatomical parameters. The retrospective design of this study did not allow for sufficient collection of echocardiography, chest imaging, and other relevant clinical data, so we were unable to adjust for the following key confounders: pulmonary hypertension, right atrial enlargement, structural or ischemic heart disease, emphysema burden, hyperinflation, hypoxemia, and cardiovascular comorbidities. Residual confounding may partially explain the observed association between chronic obstructive pulmonary disease (COPD) and P-wave axis deviation. Future research should explore that systematically collect the aforementioned data are warranted to validate and refine the findings of the present study. The COPD severity grading in this study used pre-bronchodilator FEV1% predicted values, which differs from the GOLD-recommended post-bronchodilator standard and may limit the comparability of our results with existing literature. Fourth, multiple stratified subgroup analyses by gender, age, BMI and smoking status reduced sample size within each stratum and lowered statistical power; effect sizes derived from small subgroups (especially female COPD patients *n* = 60) cannot be generalized as definitive diagnostic thresholds, and further large-sample stratified validation is necessary to confirm subgroup-specific correlations.

## Data Availability

The raw data supporting the conclusions of this article will be made available by the authors, without undue reservation.

## Footnotes

1
http://www.R-project.org
